# The Burden of Overweight and Obesity among Long-Distance Truckers in Ethiopia

**DOI:** 10.1155/2020/4242789

**Published:** 2020-07-23

**Authors:** Tewodros Yosef, Biruk Bogale, Alemnew Destaw, Angesom Weldu

**Affiliations:** Department of Public Health, College of Medicine and Health Sciences, Mizan-Tepi University, Mizan Teferi, Ethiopia

## Abstract

**Background:**

Abnormal body mass index (BMI ≥ 25 kg/m^2^) has become a major global public health problem which is rising at a faster rate in urban areas of low- and middle-income countries. In Ethiopia, the prevalence gradually increases. Long-distance truckers are at a high risk of developing overweight or obesity due to the sedentary nature of their job. Despite these populations at a high risk of developing overweight/obesity such as drivers elsewhere, pieces of data that showed the prevalence and contributing factors of overweight and obesity among long-distance truckers in Ethiopia are not yet available.

**Objective:**

To assess the prevalence and contributing factors of overweight and obesity among long-distance truckers in Ethiopia.

**Methods:**

A cross-sectional study was conducted among 400 systematically selected truckers at Modjo dry port in Ethiopia from February to March, 2018. Data were collected through face-to-face interviews using a structured questionnaire. The final results were presented in tables and numerical summary measures such as mean and standard deviation (SD).

**Results:**

Of the 400 truckers interviewed, the prevalence of overweight and obesity was 56.5%, 95% CI (51.6%–61.4%). The study also found that a monthly income ≥220 USD (AOR = 1.83, 95% CI (1.05–3.18)), having 3 or more family sizes (AOR = 2.24, 95% CI (1.15–4.36)), less than 6 hours of sleep at night (AOR = 3.34, 95% CI (1.99–5.78)), driving for 9 or more hours daily (AOR = 2.29, 95% CI (1.09–4.81)), and a truck driving experience of 10 or more years (AOR = 2.13, 95% CI (1.29–4.18)) were significantly associated with overweight and obesity.

**Conclusion:**

The prevalence of overweight and obesity was substantially high. The study also found that sociodemographic and occupational factors are mainly associated with overweight and obesity. Therefore, a health education program should be designed for awareness creation on the importance of reducing a sedentary lifestyle, consuming healthy foods or drinks, and having regular physical exercise to mitigate the problem.

## 1. Introduction

Abnormal body mass index (BMI ≥ 25 kg/m^2^) has become a major global public health problem and rising at a faster rate in urban areas of low- and middle-income countries [1, 2]. Universally, the proportion of adults with a BMI of 25 kg/m^2^ or greater increased from 28.8% to 36.9% in men and from 29.8% to 38.0% in women between 1980 and 2013 [2]. In the year 2013, 2 billion individuals worldwide were overweight or obese, and 62% of the world's obese population resided in developing countries. Overweight and obesity (abnormal BMI) are increasing even in countries with historically high levels of malnutrition [3].

Globally, abnormal BMI has more than doubled since 1980 [4]. It is associated with increased death due to a variety of noncommunicable diseases, particularly cardiovascular diseases and stroke [5]. Nearly 2.8 million people die each year because of being overweight or obese worldwide [6]. In Ethiopia, community-based studies were conducted regarding the prevalence of overweight and obesity and revealed overweight including an obesity prevalence of 30% in Addis Ababa [6] and 28.2% in Hawassa [7].

Long-distance truckers are at a high risk of developing overweight or obesity due to the sedentary nature of their job [8–12]. Overweight and obesity are dreadful problems among drivers [13–15] and are known to be risk factors for a range of diseases including hypertension, diabetes, vascular diseases, and cancers and are associated with increased crash risk and increased risk of serious or fatal injury in a crash [16–18]. Any health issue that affects drivers may result in an increased risk of road accidents [12, 19, 20].

Studies conducted worldwide regarding the prevalence of abnormal BMI revealed that 40% in Manipal, India [21], 55.5% in Chennai, India [11], 64% in central North Carolina, USA [16], 69% in South Africa [22], 71% in India [23], 75.2% in Mexico [8], 76% in Germany [24], 79.2% in Brazil [18], 91.1% in Arkansas, USA [9], and 93.3% in the USA [25]. The factors that contribute to overweight and obesity are varied and might include age, income, educational status, nutrition, sleep duration, physical exercise, daily working hours, and years of driving experience [1, 3, 7, 12, 14–16, 26–28].

Truck driving is a job that is vital to the economy of every country because manufactured goods from different parts of the world and within the country are moved to final endpoints by long- haul truckers [29]. Truckers play a fundamental role in the Ethiopian economy due to limited rail, water, and other forms of transport of goods. But, the occupational nature of truck driving predisposes drivers for long sedentary time and results in overweight/obesity [8–12]. Despite these populations being at a high risk of developing overweight/obesity, pieces of data that showed the prevalence and contributing factors of overweight and obesity among long-distance truckers in Ethiopia are not yet available. Therefore, this study aimed to assess the prevalence and contributing factors of overweight and obesity among long-distance truckers in Ethiopia.

## 2. Materials and Methods

### 2.1. Study Design, Area, and Period

A cross-sectional study was conducted from February 1 to March 1, 2018 at the Modjo dry port. The Modjo dry port is the first dry port development established in the end of 2009 to relieve the congestion of the Djibouti port. It is found in central Ethiopia, 38 miles southeast of Addis Ababa. The port handles 95% of Ethiopia's trade and the major bottleneck in Ethiopia-Djibouti trade corridor. We followed the methods of Yosef T. et al. [30].

### 2.2. Populations

The source population was all cross-country truckers at the Modjo dry port in Ethiopia. The study population was all systematically selected truckers.

### 2.3. Study Variables

The outcome variable was overweight/obesity. The independent variables were sociodemographic factors (age, monthly income, and family size), behavioral factors (chewing chat, drinking alcohol, mealtime, and physical exercise), and occupational and psychosocial factors (daily driving hours, years of truck driving, sleep hours spent at night, and perceived job stress).

### 2.4. Sample Size Determination

The minimum required sample size was calculated using a single population proportion formula, with the input of the estimated proportion of truck drivers with an abnormal body mass index (50%), precision level 5%, 95% confidence interval, and 10% for nonresponse compensation. The calculated sample size was 422.

### 2.5. Sampling Method

Except for a technical difficulty on the truck or other accident, a maximum of 15 days is required for a truck to make a round trip from Modjo dry port to the Djibouti port and back to Modjo dry port. Based on the data from the port management, an average of 300 to 400 trucks reach daily at the port. With this consideration to give each driver an equal chance of inclusion, the total sample size was divided by fifteen days and the obtained 28 truckers were studied every day. A systematic random sampling technique was used to identify potential study participants (300 trucks were divided by 28 to obtain the constant for the sampling interval, which was 11). Subsequently, taking every eleventh driver from a random start, the study was conducted until the total sample size was obtained.

### 2.6. Operational Definitions

  Body mass index (BMI) was classified as normal (BMI 18.5–24.99), overweight (BMI 25.0–29.99), and obesity (BMI ≥ 30.0) [5]
  Abnormal body mass index was defined as a BMI of ≥25 kg/m2 (overweight (BMI 25.0–29.99) and obesity (BMI ≥ 30.0))
  Prevalence was defined as the frequency of study subjects who were overweight or obese after measuring their BMI
  Cigarette smokers were defined as persons who smoked cigarettes daily, whatever be the number of cigarettes
  Proper mealtime was defined as a driver who takes his breakfast (7–9 am), lunch (12 am–2 pm), and dinner (6 pm–8 pm) based on the Ethiopian mealtime context.

### 2.7. Data Collection Instrument and Procedures

A face-to-face interview was conducted to collect the data. The interviewer-administered questionnaire comprised four sections: sociodemographic factors, behavioral factors, and occupational and psychosocial factors. First, it was made in English, translated to the local language, and then, retranslated into English to maintain its reliabilities. To calculate the body mass index of the study participants, anthropometric (weight and height) measurements were taken using a DHM-15A standardized scale (BMI height and weight body fat scale). The training was given to data collectors and supervisors regarding the objective and method of data collection and to discuss the presence of unclear questions in the questionnaire.

### 2.8. Data Processing and Analysis

The collected data were entered into EPI-data version 4.2.0.0 and, then, processed and analyzed using SPSS version 20 statistical software. Descriptive results of the independent variables were presented using frequency tables and numerical summary measures such as mean and standard deviation (SD). A binary logistic regression model was used to determine the association between the outcome variable and independent variables. The independent variables found significant with a *P* value of less than 0.25 at the bivariate level were included in the multivariable binary logistic regression model to control for potential confounding. Multicollinearity between independent variables in the model was checked, and the variance inflation factor (VIF) was found acceptable (less than 2). The Hosmer–Lemeshow goodness-of-fit test indicated (*P*=0.679) that the model was good enough to fit the data well.

## 3. Results

### 3.1. Univariate Analysis

The mean age was 37.7 (±9.13 SD) years, ranging from 22 to 59 years. The mean body mass index was 25.7 (±3.22 SD) ranging from 18.9 to 36.4 kg/m^2^. The mean daily driving duration was 11.5 (±2.76 SD) with a range of 6 to 18 hours (Table 1).

### 3.2. Body Mass Index Distribution

Of the 400 truckers interviewed, the prevalence of overweight and obesity was 194 (48.5%) and 32 (8%), respectively.

### 3.3. Distribution of Sociodemographic, Behavioral, and Occupational Factors by BMI

Of the 226 respondents with overweight or obesity, 126 (55.8%), 162 (71.7%), and 127 (56.2%) of the respondents were aged 38 years and above had 3 and more family sizes and improper mealtime, respectively. Of the 226 respondents with overweight or obesity, 195 (86.3%), 124 (54.9%), and 135 (59.7%) of truckers had a driving time of 9 and more hours daily, ≥10 years of truck driving experience, and less than 6 hours of sleep time at night, respectively (Table 2).

### 3.4. Factors Associated with Overweight and Obesity Aamong Truckers

Bivariate analysis was performed for independent variables expected to have an association with the outcome of interest. The independent variables found statistically significant at a *P* < 0.25 in the bivariate analysis were included in the multivariable binary logistic regression model. The multivariable binary logistic regression model was performed, and independent variables found associated with the outcome of interest at a *P* value of less than 0.05 were declared significant. According to the multivariable binary logistic regression model, a monthly income ≥220 USD (AOR = 1.83, 95% CI (1.05–3.18)), having 3 or more family sizes (AOR = 2.24, 95% CI (1.15–4.36)), less than 6 hours of sleep at night (AOR = 3.34, 95% CI (1.99–5.78)), driving for 9 or more hours daily (AOR = 2.29, 95% CI (1.09–4.81)), and driving experience of 10 or more years of truck (AOR = 2.13, 95% CI (1.09–4.18)) were significantly associated with overweight and obesity (Table 3).

## 4. Discussion

Several studies have shown that long-distance truckers are at a high risk of developing overweight or obesity due to the sedentary nature of their job [8–12]. Based on the abovementioned scenario, we aimed to assess the prevalence and contributing factors of overweight and obesity among long-distance truckers in Ethiopia. After collecting and analyzing the data, the prevalence of overweight and obesity was 56.5%, 95% CI (51.6%–61.4%). This study was in line with 55.5% in Chennai, India [11]. It was higher than 40% in Manipal, India [21]. But, it was far lower than 64% in central North Carolina, USA [16], 69% in South Africa [22], 71% in India [23], 75.2% in Mexico [8], 76% in Germany [24], 79.2% in Brazil [18], 91.1% in Arkansas, USA [9], and 93.3% in the USA [25]. The variation observed is explained by the difference in socioeconomic and behavioral factors. Most of the higher prevalence studies were conducted in areas where fast-fried foods, sweetened foods, and sweetened soft and energy drinks are frequently consumed than the current study.

The study revealed that the monthly income of drivers was significantly associated with the occurrence of overweight/obesity. Truckers with a monthly income of 220 USD or more were 1.8 times more likely of develop overweight/obesity. Previous conducted studies also support the positive association between income and overweight/obesity [1, 7]. This could be due to more income associated with more intake of foods and drinks because of the capacity to purchase at any time. The associated family size and overweight/obesity were also revealed in this study. Those drivers with family sizes of 3 or more were 2.2 times more likely to develop overweight/obesity than those drivers with fewer than 3 family sizes. This could be due to stress as a result of thinking extremely about how to feed their families. This makes them drive long distances (to make the frequent round trip) to get more money and results in a long sedentary time in front of the steering wheel.

Truckers with less than 6 hours of sleep at night were 3.3 times more likely of developing overweight/obesity than those with 6 or more hours of sleep. As a result, the very strong association between sleep duration and overweight/obesity was revealed in this study. This finding was consistent with previously conducted studies [3, 12, 15].

Of the 226 truckers with overweight/obesity, 195 (86.3%) truckers were driving for 9 or more hours per day. As a result, this study showed the presence of an association between daily driving hours and overweight/obesity. Truckers with 9 or more hours of driving daily had 2.3 times increased odds of developing overweight/obesity than driving less than 9 hours daily. This finding was consistent with studies conducted in other areas [15, 16, 27]. This could be the long sedentary time that resulted from long driving hours because long sedentary time is associated with the occurrence of overweight/obesity [1, 7, 12, 14].

Of the 226 truckers with overweight/obesity, 124 (54.9%) of the truckers had 10 or more years of driving experience. Truckers with 10 or more years of truck-driving experience had twice the odds of developing overweight/obesity than truckers with less than 10 years of driving experience. The study showed an association between years of driving experience and overweight/obesity. This finding was consistent with previously conducted studies in other areas [14, 26].

## 5. Conclusions

The prevalence of overweight and obesity was substantially high. The study also found that sociodemographic and occupational factors are mainly associated with overweight and obesity. Therefore, a health education program should be designed for awareness creation on the importance of reducing sedentary lifestyle, consuming healthy foods or drinks, and having regular physical exercise to mitigate the problem.

## Figures and Tables

**Table 1:** Descriptive statistics of continuous variables among long-distance truckers in Ethiopia.

Variables	Mean ± SD
Age (years)	37.7 ± 9.13
Monthly income (USD)	220 ± 91
Weight (kilogram)	75.4 ± 10.5
Height (centimeter)	171.2 ± 6.99
Body mass index (kg/m^2^)	25.7 ± 3.22
Daily driving duration (hours)	11.5 ± 2.76

**Table 2:** Distribution of sociodemographic, behavioral, and occupational factors by BMI among long-distance truckers in Ethiopia (*n* = 400).

Variables	Categories	Body mass index	
		Normal, *n* (%)	Overweight/obese, *n* (%)
Age	<38 years	103 (59.2%)	100 (44.2%)
	≥38 years	71 (40.8%)	126 (55.8%)
Monthly income	<220 USD	99 (56.9%)	101 (44.7%)
	≥220 USD	75 (43.1%)	125 (55.3%)
Family size	<3	70 (40.2%)	64 (28.3%)
	≥3	104 (59.8%)	162 (71.7%)
Smoking cigarettes	Yes	46 (26.4%)	78 (43.8%)
	No	128 (73.6%)	148 (56.2%)
Mealtime	Proper	109 (62.6%)	99 (43.8%)
	Improper	65 (37.4%)	127 (56.2%)
Daily driving duration	<9 hours	48 (27.6%)	31 (13.7%)
	≥9 hours	126 (72.4%)	195 (86.3%)
Years of truck driving	<10 years	109 (62.6%)	102 (45.1%)
	≥10 years	65 (37.4%)	124 (54.9%)
Sleep hours spent at night	<6 hours	61 (35.1%)	135 (59.7%)
	≥6 hours	113 (64.9%)	91 (40.3%)

**Table 3:** Factors associated with overweight and obesity among long-distance truckers in Ethiopia (*n* = 400).

Variables	Categories	COR (95% CI)	AOR (95% CI)	*P* value
Age	<38 years	1	1	
	≥38 years	1.83 (1.23–2.73)^*∗∗*^	0.94 (0.46–1.90)	0.862
Monthly income	<220 USD	1	1	
	≥220 USD	1.63 (1.10–2.43)^*∗*^	1.83 (1.05–3.18)	**0.032**
Family size	<3	1	1	
	≥3	1.70 (1.12–2.59)^*∗*^	2.24 (1.15–4.36)	**0.017**
Smoking cigarettes	Yes	1.47 (0.95–2.26)^*∗*^	1.41 (0.84–2.37)	0.189
	No	1	1	
Mealtime	Proper	1	1	
	Improper	2.15 (1.44–3.22)^*∗∗*^	1.49 (0.89–2.49)	0.126
Sleep hours spent at night	<6 hours	2.75 (1.83–4.14)^*∗∗*^	3.34 (1.99–5.78)	**<0.001**
	≥6 hours	1	1	
Daily driving hours	<9 hours	1	1	
	≥9 hours	2.40 (1.45–3.97)^*∗∗*^	2.29 (1.09–4.81)	**0.029**
Years of truck driving	<10 years	1	1	
	≥10 years	2.04 (1.36–3.05)^*∗∗*^	2.13 (1.29–4.18)	**0.028**

CI = confidence interval, COR = Crude odds ratio, AOR = adjusted odds ratio. ^*∗*^ = Significant at a *P* value <0.25, ^*∗∗*^ significant at a *P* value < 0.01.

## Data Availability

The datasets used and/or analyzed during the current study are available from the corresponding author upon reasonable request.

## Abbreviations

AOR: Adjusted odds ratio
BMI: Body mass index
CI: Confidence interval
COR: Crude odds ratio
SPSS: Statistical package for the social sciences
SD: Standard deviation
USA: United States of America
USD: United States Dollar.
